# *ANRIL*: A lncRNA at the CDKN2A/B Locus With Roles in Cancer and Metabolic Disease

**DOI:** 10.3389/fendo.2018.00405

**Published:** 2018-07-24

**Authors:** Yahui Kong, Chih-Heng Hsieh, Laura C. Alonso

**Affiliations:** Department of Medicine, Diabetes Center of Excellence, University of Massachusetts Medical School, Worcester, MA, United States

**Keywords:** *ANRIL*, *CDKN2A*, *CDKN2B*, long noncoding RNA, diabetes, pancreatic islet, cancer, metabolic disease

## Abstract

The *CDKN2A/B* genomic locus is associated with risk of human cancers and metabolic disease. Although the locus contains several important protein-coding genes, studies suggest disease roles for a lesser-known antisense lncRNA encoded at this locus, called *ANRIL*. *ANRIL* is a complex gene containing at least 21 exons in simians, with many reported linear and circular isoforms. Like other genes, abundance of ANRIL is regulated by epigenetics, classic transcription regulation, splicing, and post-transcriptional influences such as RNA stability and microRNAs. Known molecular functions of *ANRIL* include *in cis* and *in trans* gene regulation through chromatin modification complexes, and influence over microRNA signaling networks. Polymorphisms at the *ANRIL* gene are linked to risk for many different cancers, as well as risk of atherosclerotic cardiovascular disease, bone mass, obesity and type 2 diabetes. A broad array of variable reported impacts of polymorphisms on *ANRIL* abundance, splicing and function suggests that *ANRIL* has cell-type and context-dependent regulation and actions. In cancer cells, *ANRIL* gain of function increases proliferation, metastasis, cell survival and epithelial-mesenchymal transformation, whereas *ANRIL* loss of function decreases tumor size and growth, invasion and metastasis, and increases apoptosis and senescence. In metabolic disease, polymorphisms at the *ANRIL* gene are linked to risk of type 2 diabetes, coronary artery disease, coronary artery calcium score, myocardial infarction, and stroke. Intriguingly, with the exception of one polymorphism in exon 2 of *ANRIL*, the single nucleotide polymorphisms (SNPs) associated with atherosclerosis and diabetes are non-overlapping. Evidence suggests that *ANRIL* gain of function increases atherosclerosis; in diabetes, a risk-SNP reduced the pancreatic beta cell proliferation index. Studies are limited by the uncertain relevance of rodent models to *ANRIL* studies, since most *ANRIL* exons do not exist in mouse. Diverse cell-type-dependent results suggest it is necessary to perform studies in the relevant primary human tissue for each disease. Much remains to be learned about the biology of *ANRIL* in human health and disease; this research area may lead to insight into disease mechanisms and therapeutic approaches.

## Introduction

The discovery of functional noncoding RNAs has opened a kaleidoscopic world of unanticipated mechanisms extending far beyond the DNA-RNA-protein paradigm; noncoding RNAs may in fact outnumber coding RNAs (1). Long noncoding RNAs (lncRNAs) have been discovered throughout the genome; scientists are working to explore their functions in health and disease. The *ANRIL* lncRNA was first identified in a melanoma kindred with a large (403 kb) deletion at the *CDKN2A/B* locus (2). *ANRIL* has attracted broad attention because it is located at a genomic hotspot for disease heritability, the *CDKN2A/B* locus. Although protein coding genes at this locus have important well-studied roles in cell cycle regulation, data suggest that some locus disease-associated single nucleotide polymorphisms (SNPs) act through effects on *ANRIL* itself. Intriguingly, studies suggest *ANRIL* not only impacts the biology of cancer, but also has cell-type-specific roles in metabolic disease. Although *ANRIL* has been reviewed in the past (3, 4), knowledge has exponentially increased in recent years. Here we review advances in ANRIL SNPs, gene regulation, cell biology, and disease roles of *ANRIL*.

## The *CDKN2A/B* locus

**Figure 1:**
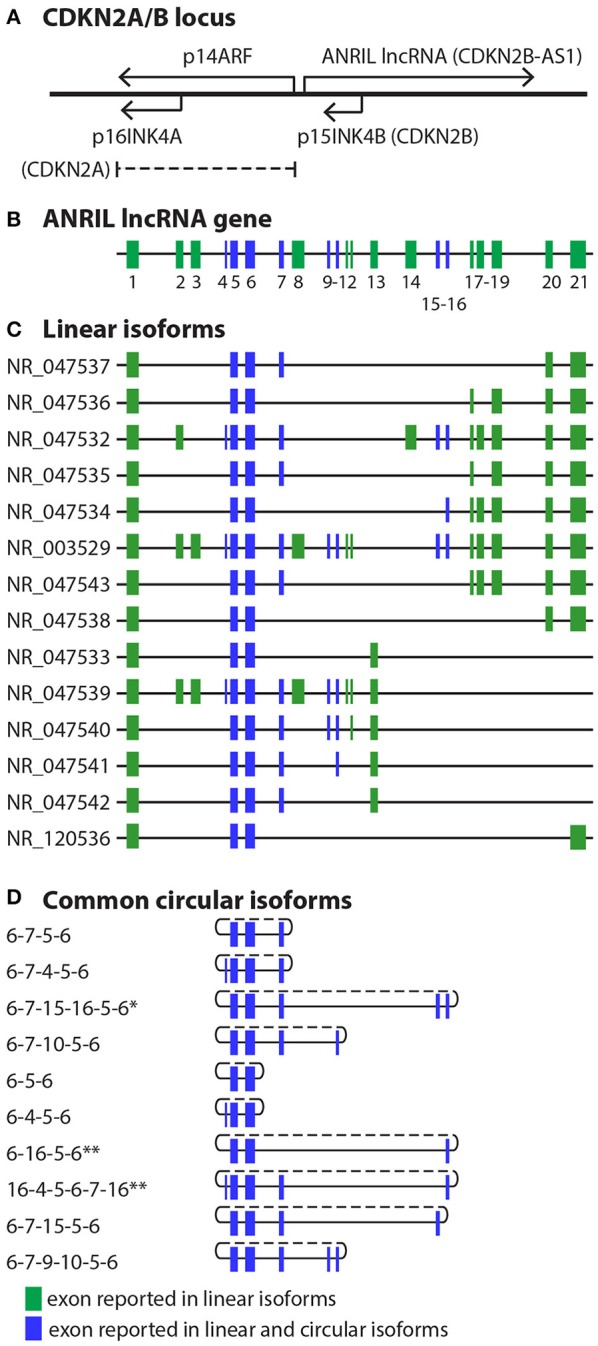
CDKN2A/B locus and *ANRIL* isoforms. **(A)** At the CDKN2A/B locus, the *ANRIL* lncRNA is antisense to the protein coding genes. The p15INK4B gene is contained within intron 1 of *ANRIL*. **(B)** To date, *ANRIL* has 21 reported exons. **(C,D)** Numerous linear **(C)** and circular **(D)** exons have been reported. Due to the discovery of additional exons, distal exons in some circular isoforms have been renumbered based on the current 21 reported exons. ^*^Exons 15-16 refer to exons 14-15 in Holdt et al. (6). ^**^Exon 16 refers to exon 14 in Sarkar et al. (7) and Burd et al. (8).

*ANRIL*, or *CDKN2B-AS1*, is located at the human *CDKN2A/B* locus at 9p21.3. This gene cluster, extending over a nearly 350 kb genomic region housed within a single topologically associated domain (TAD) (5), contains three protein coding genes and, antisense to them, the *ANRIL* lncRNA (Figure 1). The protein coding genes include *S-methyl-5*′*-thioadenosine phosphorylase* (*MTAP*), *CDKN2A*, which encodes splice variants p16^INK4A^ and p14^ARF^, and *CDKN2B*, which encodes p15^INK4B^ (9, 10). *MTAP* lies at one end of the locus, 192 kb telomeric to the 5′ start of *ANRIL*. At the centromeric end of the locus, the *ANRIL* gene contains 19–21 reported exons over a 126 kb region. *CDKN2A* lies between *MTAP* and *ANRIL*, near the first exon of *ANRIL*; *CDKN2B* is located within the first intron of *ANRIL*, in an antisense direction. The proteins encoded by *CDKN2A* and *CDKN2B* are tumor suppressors with well-established roles in cell proliferation, apoptosis, senescence and aging (11, 12). p16^INK4A^ and p15^INK4B^ are cyclin dependent kinase (CDK) inhibitors, inhibiting retinoblastoma phosphorylation by CDK4/6. The p14^ARF^ protein, a splice variant of *CDKN2A* which due to a frame shift has no amino acid homology to the principal other *CDKN2A* splice variant, p16^INK4A^, modulates p53 activity. *ANRIL* is transcribed by RNA polymerase II and spliced into multiple linear and circular isoforms in a tissue-specific manner. In general, *ANRIL* roles, explored in detail below, include gene regulation in *cis* and in *trans* through interaction with polycomb repressive complex (PRC) histone modifiers, as well as RNA-RNA interactions such as microRNA (miRNA) sponge activity (3, 13). Known biological impact of *ANRIL* activities include modulation of proliferation, apoptosis and cellular adhesion pathways (14).

## Evolution of the *ANRIL* gene

The evolutionary development of the human *ANRIL* gene has been studied by comparative analysis of the genomes of 27 organisms including non-mammalian vertebrates, non-placental mammals, non-primate placental mammals, and primates (15). *ANRIL* originated in ancestors of the Eutherian (placental) mammalian clade. Initially the gene contained only a few exons; over time, *ANRIL* underwent clade-specific evolution, adding exons in many mammals but losing exons in rodents. The full 21 exon gene is present only in simians. *ANRIL* genes contain many repeat elements, both intronic, and exonic; evidence suggests that transposon activity has mediated many of the observed evolutionary changes in exon presence or absence, location, sequence, conservation, and structure, as well as introduction of splice sites (15).

Early *ANRIL* variants were likely not transcribed or functional (15). One hallmark of functional RNAs is splice signals at intron/exon boundaries. In simians, 191 intron/exon boundaries contained canonical splice signals, while 20 did not. In lower mammals, however, only about half of intron/exon junctions contained identifiable splice signals (15). This finding suggests that as *ANRIL* gained exons, and exon sequences became more conserved across species, it also increased the number of splice signals and gained functionality. Taken together, data suggest that *ANRIL* may be functional only in simians, and that functionality may have been introduced by transposon activity (15).

## *ANRIL* isoforms and structure

With at least 21 exons (new exons discovered as recently as 2017 (7)), the *ANRIL* gene can potentially generate a large number of splice variants. In fact, many *ANRIL* isoforms have been reported (Figure 1) (16). Exon numbering has changed over time as new exons were discovered. Studies observe multiple isoforms in any given cell type, mostly at low abundance. A different range of isoforms may be identified from one cell type to another, but tissue-dependent isoform expression in primary cells or tissues has not yet been comprehensively quantified using the same reagents and techniques. Intriguingly, many studies have now identified both linear and circular *ANRIL* isoforms (6–8). The longest open reading frame identified in any *ANRIL* variant is 86 codons, supporting the concept that functionality of this gene is through RNA activity (16).

### Linear and circular isoforms

Many conventional linear polyadenylated *ANRIL* isoforms are detected in different cell types. Circular *ANRIL* (circ*ANRIL*) isoforms, without polyadenylation, have also been described. Circular RNAs, which are formed by “back-splicing” in which a downstream splice donor site is joined to an upstream splice acceptor site, were discovered in 2012 to be a broadly occurring phenomenon across developmental stages and tissues, arising from at least 14% of human transcribed genes (17, 18). CircRNAs enjoy distinct properties from linear RNAs, including, in general, enhanced stability and longevity, cytoplasmic localization, and lack of translation (although if an IRES is engineered, circRNAs can support translation) (19). Traditional PCR using antisense-oriented primers cannot distinguish between linear and circular isoforms; other methodology, such as PCR using “outward-facing” primers directed away from each other, detection of specific exon-exon junctions, or protection from RNAse R digestion can quantify circular RNAs.

Careful examination of melanoma cell lines showed that the abundance of individual *ANRIL* exons is non-uniform, supporting the presence of different isoforms (7). In both transformed cell lines and in human brain derived cells, abundance of *ANRIL* exons was lower than exons from locus protein-coding genes *CDKN2A* and *CDKN2B* (8). Linear isoforms tend to include proximal exons (1-2), whereas isoforms with only central exons (4-16) are more likely to be circular (8). In melanoma cells, proximal exons (exon 1 and exon 5-6) were more highly expressed than distal exons. This suggests that short isoforms of *ANRIL*, which tend to include proximal exons, are more abundant than longer isoforms in this cell type (7). In human peripheral blood mononuclear cells and a monocyte cell line, four major groups of *ANRIL* transcripts were found, all with common proximal exons including exons 1, 5, and 6 but with different distal exons, of various lengths (8). Multiple circular *ANRIL* isoforms have been detected. A circ*ANRIL* isoform with an exon 14-5 head-to-tail junction was reported to be the predominant form in both an immortalized fibroblast cell line (8) and in a majority of melanoma cell lines (7). Other non-canonical back-spliced junctions observed in melanoma samples included exon 14-5, 7-4, 10-5, and 14-4 (7). The exons most commonly observed in circ*ANRIL* in melanoma cells were 4, 5, 6, 7, 10, 13, and 14; in varied human cell types, the majority of circ*ANRIL* species were exon 5-6-7 containing (6). *ANRIL* exons 1, 2, 3, 8, 9, 11, and 12 were rarely included in circular RNA products (8). In melanoma lines, no correlation was observed between abundance of linear and circ*ANRIL* (7). However, circ*ANRIL* expression was inversely correlated with linear *ANRIL* expression in peripheral blood mononuclear cells in a cardiovascular cohort (6). Circ*ANRIL* was found to be resistant to RNAse R digestion compared with linear *ANRIL*, and an actinomycin D time course confirmed enhanced stability of the 14-5 circ*ANRIL* isoform compared with linear isoforms (7, 8).

### Secondary structure

Structure and function of lncRNAs is of high interest in the scientific community, given the increasing recognition of lncRNA roles in cancer and the normal biology of higher organisms. As such, prediction of lncRNA structures is an important computational challenge. One approach is to identify structural elements through comparison of related lncRNAs. The MONSTER tool was used to compare *ANRIL* to two lncRNAs with similar biological function: *HOTAIR* and *COLDAIR* (20). MONSTER identifies sequence-predicted secondary structure, such as regions likely to be single stranded RNA, double stranded RNA, hairpin loops, interior loops and bulges. Comparing predictions of two lncRNAs with similar functions is proposed as a mechanism to identify structural motifs. When *HOTAIR, COLDAIR*, and *ANRIL* were compared, several common structures were identified, putative structural motifs related to their common function in epigenetic regulation, which could lead to a molecular understanding of mechanism of action in future studies (20). Another study identified the region of ANRIL that interacts with CBX7, a polycomb repressor component; secondary structure analysis revealed hairpin structural motifs with significant binding affinity to CBX7. Fluorescence anisotropy suggested a ternary complex between a particular loop of ANRIL, CBX7 and a H3K27me3 methylated histone peptide (21).

### Cellular localization

RNA localization impacts function. In melanoma cells, linear *ANRIL* species containing proximal (exon 1) and distal (exons 13b, 19) exons were predominantly found in the nucleus. However, middle exons (exons 5, 6, and 7), which are found in both linear and circ*ANRIL*, were observed in cytoplasmic fractions, suggesting that *circANRIL* species may be predominantly cytoplasmic (7). Nuclear localization suggests linear isoforms may be responsible for the known *ANRIL* function of regulating gene transcription via chromatin modulation (see below). Conversely, cytoplasmic localization suggests *circANRIL* forms may participate in post-transcriptional functions. In gastric (22), prostate (21), and urothelial (23) cancer cells, ANRIL was predominantly nuclear. In a beautiful high-resolution analysis of single-molecule lncRNA localization, ANRIL was found to be mostly localized to cell nuclei, in one or several bright foci. Like other lncRNAs analyzed, ANRIL nuclei foci were lost in mitotic cells (24). Physiological stimuli that change ANRIL localization may provide clues as to ANRIL functions. Intriguingly, in a retinal cell line *ANRIL* isoforms were observed by fluorescence in situ hybridization to localize to the peri-nuclear cytoplasmic space. *ANRIL* abundance was induced by glucose, but *ANRIL* localization did not change with high glucose exposure (25). On the other hand, a study in HUVEC cells, using primers predicted to detect both linear and circular isoforms, found *ANRIL* to be mostly nuclear; nuclear *ANRIL* was increased after exposure to TNF-α (26). At least one study has used *ANRIL* as a nuclear positive control to test localization of other transcripts (27). Future cell type specific studies of ANRIL localization under basal, stimulated, and stress conditions may lead to clues as to ANRIL roles in tissue health and disease.

## Regulation of *ANRIL* abundance

**Figure 2:**
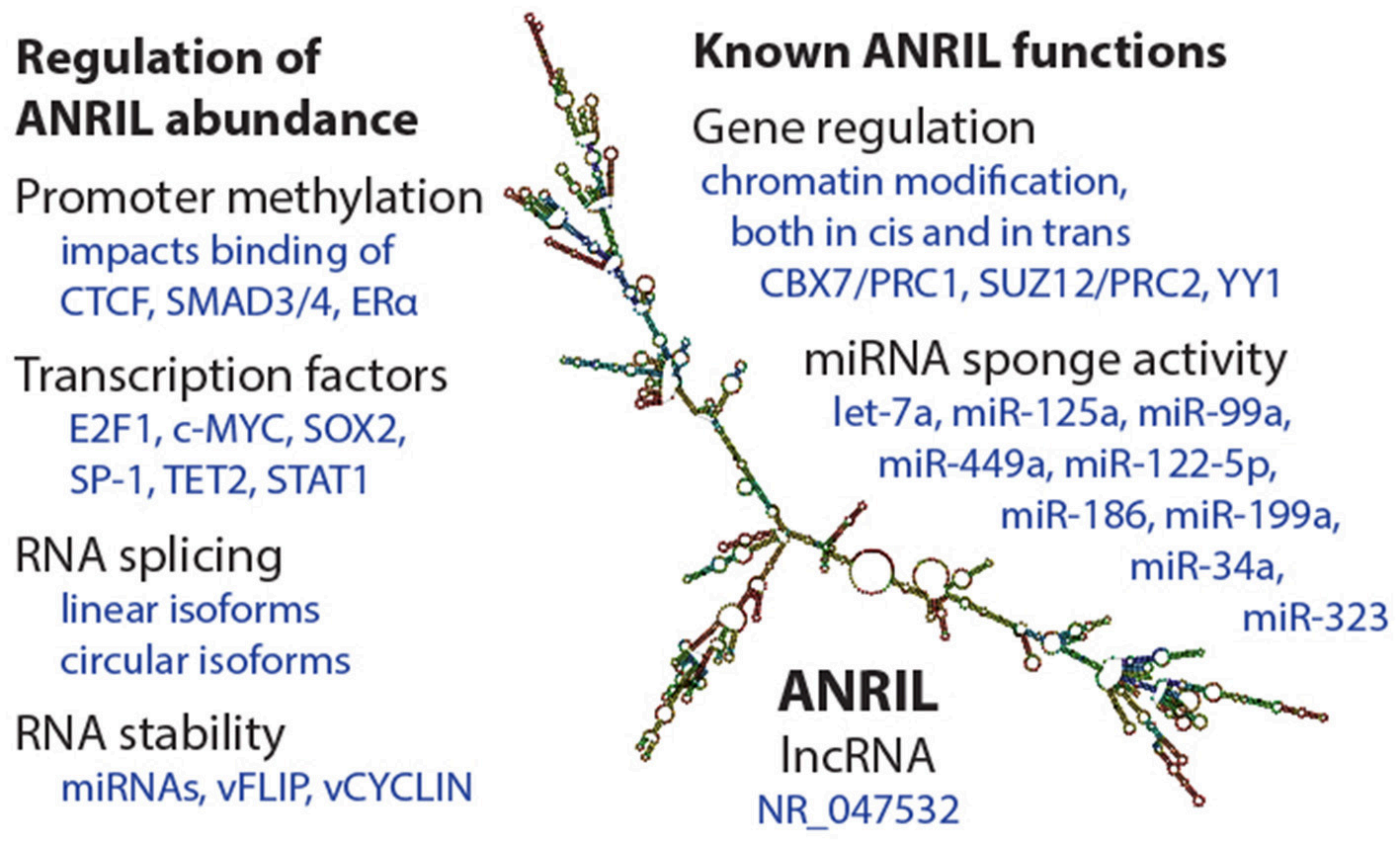
Summary of regulation and functions of the ANRIL lncRNA. **(Left)** Some of the known mechanisms by which ANRIL abundance is regulated, at the transcriptional and post-transcriptional levels. On the **(Right)**, a selection of known ANRIL cellular functions are depicted. We apologize for observations not included in this summary image. The ANRIL structural prediction in the center is of a common long-isoform of ANRIL, and was generated from Gruber et al. (28).

Abundance of *ANRIL* species is determined by promoter transcriptional activity, splicing decisions, and RNA stability (Figure 2). Like other genes, *ANRIL* promoter activity is influenced by epigenetic control and transcription factor occupancy. Intriguingly, epidemiological findings suggest that epigenetic regulation of *ANRIL*, through promoter methylation, has important long-lasting consequences for tissue function (29–31). As such, *ANRIL* regulation is one mediator of the impact of early life environmental signals on adult human health.

### *ANRIL* promoter methylation

Epidemiological and experimental findings demonstrate that methylation of the *ANRIL* promoter region regulates *ANRIL* gene expression and has functional importance. The first exons of *ANRIL* and *p14ARF* are separated by only 300 bp, in head-to-head antisense orientation; the intergenic region between them is a bidirectional promoter (4, 32, 33). *In silico* analysis of ENCODE ChromHMM data (34) revealed that this region is enriched for both promoter and enhancer activity, and DNAse I hypersensitivity, across multiple cell types, suggesting this is a regulatory region. This region is bound by CTCF, usually considered to be a transcriptional repressor, insulating promoters from enhancer activity. Oddly, CTCF binding at the *ANRIL* promoter was associated with active-chromatin mark histone H3K4 trimethylation (35). CTCF binding, and *ANRIL* and p14ARF expression, were inhibited by methylation of local CpG islands and increased by demethylation. Knockdown of CTCF prevented the demethylation-induced expression of *ANRIL* and p14ARF, confirming that CTCF is a methylation-sensitive positive regulator of *ANRIL* promoter activity (35).

Additional evidence supports the functional importance of CpG sites for *ANRIL* promoter activity, locus gene expression and transcription factor binding. Mutagenesis of the CpG sites affects both *ANRIL* and p14ARF promoter activity (29, 30). Methylation status of several CpG differentially methylated regions at *ANRIL* in umbilical cord tissues was positively associated with abundance of linear but not circular *ANRIL*, but inversely associated with p14ARF and p16INK4a expression (29). CpG methylation also affects other transcription factors binding at the *ANRIL* promoter to regulate downstream gene expression, such as interferon gamma, SMAD3/4 and ERα (29, 30). Methylation status of CpG islands around the *p16INK4A* transcription start site was also shown to coordinate transcription of *ANRIL* and p16INK4A in human cells (36). Given the multiple protein complexes binding across different CpG sites, and variable quantitative impact of individual CpG region mutagenesis on *ANRIL* isoforms and locus gene expression, regulation appears to be complex.

### Transcription factors regulate *ANRIL* production

*ANRIL* expression is influenced by cellular processes such as genotoxic stress, tumorigenesis, senescence, and inflammation. Activity at the bidirectional promoter region upstream of both *ANRIL* and *p14ARF* genes is influenced by the critical cell cycle regulator E2F1 (32, 33). In response to the genotoxic stress of DNA damage, E2F1 transcriptionally activates *ANRIL* in an ATM-dependent manner (33, 37). In this case, *ANRIL* is thought to promote cell growth by suppressing locus INK-family inhibitors after DNA repair is complete, allowing re-entry into cell cycling (33). The *ANRIL* promoter was also responsive to E2F1 in cancer cells (32). In addition to E2F1, several known potent oncogenes regulate *ANRIL* expression in various cancers. In lung cancer, c-MYC binds to an E-box in the *ANRIL* promoter and induces *ANRIL* expression (38). In nasopharyngeal carcinoma, transcription factor SOX2 was shown to bind directly to the *ANRIL* promoter and activate transcription of *ANRIL* and its downstream effector β-catenin (39). In liver cancer, SP1 binds the *ANRIL* promoter and positively regulates *ANRIL* transcription (40). On the other hand, TET2, a tumor suppressor in human gastric cancer, binds to the promoter region of *ANRIL* and regulates expression of *ANRIL* as well as p16INK4a, p15INK4b, and p14ARF (41). Transcription regulation of *ANRIL* is involved not only in cell DNA damage and oncogenesis, but also in disparate processes such as cell senescence and inflammation. In senescence, oncogenic Ras was found to reduce expression of *ANRIL* (13, 21, 42). In inflammation, STAT1 activates the *ANRIL* locus in vascular endothelial cells has been reported; CAD-associated *ANRIL* SNP rs10757278, located in a known downstream enhancer region, disrupts the STAT1 binding site and modulates IFN-γ induced *ANRIL* expression via stimulation (43). Intriguingly, the binding of STAT1 at this enhancer exerts cell-type specific regulation of *ANRIL* expression: repression in lymphoblastoid cells lines, but activation in HUVEC cells (43). In sum, data support an important role for cell-type specific transcriptional regulation of the *ANRIL* lncRNA in a range of cellular processes and outcomes.

### Regulation of *ANRIL* splicing

Cell type dependent variation in abundance of different *ANRIL* isoforms suggests that splicing may be a point of regulation (8, 44). Almost nothing is known about *ANRIL* splicing decisions. Disease-associated *ANRIL* gene polymorphisms have shed light on this process. In lymphocytes, the coronary artery disease (CAD) associated SNP rs10757278 (intron 12) correlates with abundance of certain circular (14-5 and 4-6) and linear (exon 1-2, but not 18-19, containing) isoforms (8). The rs10757278 A allele was found to inhibit skipping of exon 15, promoting circ*ANRIL* species ending in exon 14 (8). Mechanisms regulating *ANRIL* splicing require further study.

### Post-transcriptional regulation

Determinants of *ANRIL* transcript longevity and stability remain uncertain, but miRNAs can participate. *ANRIL*, downregulated following Kaposi's sarcoma associated herpesvirus (KSHV) infection, contains multiple seed matches for KSHV miRNAs. Forced miRNA expression decreased *ANRIL* abundance, and miRNA pull-down experiments confirmed a direct interaction. In addition, KSHV latency associated proteins vFLIP and vCyclin also decreased *ANRIL* abundance, suggesting post-transcriptional miRNA-dependent and independent regulation (45).

## Functions of the *ANRIL* lncRNA

### Transcription regulation via chromatin modifying complexes

Many studies show that ANRIL functions in cells to regulate gene expression via chromatin modification. Acting *in cis, ANRIL* interacts with both PRC-1 and−2 to mediate epigenetic transcriptional repression of neighboring genes *CDKN2A* and *CDKN2B*, through mechanisms involving histone modification and chromatin remodeling (13, 21, 33). *ANRIL* interacts with PRC1 component CBX7 to recruit PRC1 to the p14ARF and p16INK4A loci, silencing the *CDKN2A* locus by H3K27-trimethylation (21). At *CDKN2B, ANRIL* was shown to recruit SUZ12, a subunit of the PRC2 (13). *ANRIL* also interacts with PRC-associated protein YY1 (46). Intriguingly, the structural conformation of the methyl-lysine binding pocket in the chromodomain of CBX7, which interacts with H3K27-trimethylation to cause chromatin compaction, is influenced by allosteric RNA-protein binding with *ANRIL* (47). However, despite this well-documented repression of other locus genes by *ANRIL*, a positive correlation between *ANRIL* (both short and long isoforms), *CDKN2A* and *CDKN2B* RNA abundance has been frequently reported, suggesting transcriptional co-regulation of these genes predominates in many tissues (8, 10, 14, 16, 44, 48–51).

*ANRIL* also acts in a PRC1/2 dependent mechanism to repress distant genes *in trans* (32, 46). Trans regulation by *ANRIL* may be dependent on Alu motifs, which are found both in *ANRIL* transcripts and in the promoters of *ANRIL* target genes (46). This mechanism was shown to regulate the CARD8 gene in endothelial cells (52). Polycomb group proteins, which are highly enriched near Alu motifs across the genome, are recruited to target gene promoters upon *ANRIL* over-expression. In support of this concept, silencing *ANRIL* impacts expression of a large number of genes across the genome (14). Separate from chromatin modification, *ANRIL* is reported to regulate Wnt signaling by binding to SOX2, increasing transcriptional activity of the WNT/β-catenin pathway (39).

### miRNA abundance and activity

*ANRIL* also influences gene expression via miRNA networks. *ANRIL* regulates miRNAs both at the epigenetic level, through regulation of miRNA transcription, and through direct binding to miRNAs, acting as a miRNA “sponge.” In gastric cancer cells, *ANRIL* epigenetically silences miR-99a/miR-449a through a PRC2 mechanism (22). In general, expression of *ANRIL* and its target miRNAs are negatively correlated in tissues and cell lines (22, 53–57). *ANRIL* has been described as having pro-oncogenic effects by sponging miRNAs (see below for more details). On the other hand, circ*ANRIL* containing exons 5-6-7 was found to lack miRNA sponge activity (6). Inhibition of miRNAs can reverse the effects of *ANRIL* knockdown.

### Cellular outcomes of *ANRIL* activity

*ANRIL* has broad impacts on cell biology, including influence over proliferation, senescence, apoptosis, extracellular matrix remodeling, and inflammation (14). In cancer, *ANRIL*-miRNA interactions regulate networks of downstream targets of miRNAs, promoting an oncogenic role for *ANRIL* in cell proliferation, metastasis, invasion, radio-resistance, drug-induced cytotoxicity and apoptosis, involving many different signaling pathways (22, 53–57). Specifically, repression of cell cycle inhibitors p14ARF, p15INK4B, and p16INK4A increases proliferation, decreases senescence, and contributes to the DNA damage response (13, 21, 33). PRC-mediated epigenetic repression of Kruppel-like factor 2 (KLF2) influences proliferation and apoptosis (40, 58). Cooperation between *ANRIL* and PRC-associated YY1 increases TNF-alpha dependent inflammatory mediators (IL-6, IL-8) through NF-kB (26). *ANRIL* influences the cellular response to oxidative stress through a miR-125a regulation of MCL-1 (59). Circular *ANRIL* species were found to regulate ribosome biogenesis in vascular smooth muscle cells (6).

## The *ANRIL* gene is associated with human disease

A primary driver of interest in *ANRIL* is the large body of genomic data linking the *ANRIL* gene with risk of human disease. Genome-wide association studies (GWAS) have identified many disease-associated SNPs in or near the *ANRIL* gene (60). The *CDKN2A/B* locus is remarkable for the large number of associated diseases, ranging from aging and frailty to cancer to metabolic disease. Perhaps surprisingly given the validated importance of the products of the *CDKN2A* and *CDKN2B* genes in cell biology, in some cases *ANRIL* expression shows stronger phenotype association than protein-coding *CDKN2A/B* locus genes (4, 48), linking *ANRIL* itself to a range of important human diseases.

### *ANRIL* SNPs and disease risk

Studies indicate that SNPs in the ANRIL gene can impact ANRIL expression and function. The *CDKN2A/B* locus is associated with risk of cancer, atherosclerotic disease, type 2 diabetes, stroke, aneurysm, periodontitis, Alzheimer's disease, aging, frailty, glaucoma, endometriosis, multiple sclerosis, hypertension (10, 61). Reviewed here are only SNPs within or downstream of the *ANRIL* gene; broader *CDKN2A/B* locus disease associations have been reviewed previously (10, 62). Integrating information from published observations and the NCBI linkage disequilibrium database (63), we find that disease-associated SNPs in the *ANRIL* gene that modulate locus gene expression fall into approximately six groups (Table 1) defined loosely by linkage block and reported effects. Exceptions outnumber the rules, however; for nearly all groups there are reports of SNPs with different or even opposite effects. Summarized here is a generalized synopsis of the majority of reports. Group A SNPs, while located in *ANRIL* introns, generally impact *CDKN2A/B* but not *ANRIL* biology (48, 50, 64). All other SNP groups have reported impacts on *ANRIL* itself, but reports often describe conflicting direction of change. Some SNPs are reported to fall in enhancer regions (43, 49, 64) or to impact *ANRIL* splicing (8) or secondary structure (69, 74). The data are incomplete. A particular weakness of the field is that although tissue-specific effects are likely to determine how polymorphisms impact disease risk, in many cases the relevant primary tissue has not been tested.

**Table 1:** Disease associated SNPs in/near the *ANRIL* gene that modulate locus gene expression.

**SNP group**	**Location in *ANRIL***	**SNPs**	**Diseases associated**	**Cell type tested**	**Impact**	**References**
A	Intron 1	rs2811712 rs598664 rs3218018 rs3218005	Frailty, cancers, diabetes, MI, CAC	Blood, leukocytes	Altered *CDKN2A* and *CDKN2B* expression, but no change in *ANRIL* expression or not reported	(14, 48, 50, 64, 65)
	Intron 2	rs662463				
B	Intron 1	rs3217992 rs3218020	CAD, glaucoma, cancer	Blood	Risk SNPs decrease *ANRIL* expression	(14, 48)
C	Intron 1	rs1063192	CAD, glaucoma, stroke, MI, diabetes, cancers	Blood, lympho-blastoid cells, HUVEC, lymphocytes, islets	Increase/decrease *ANRIL* expression. Possible enhancer. Disrupt miRNA binding site. Reduce beta cell proliferation index	(14, 48, 66–68)
	Exon 2	rs564398				
	Intron 2	rs7865618				
D	Intron 1	rs7044859 rs496892	Cancers, CAD, Stroke, MI, CAC, glaucoma, cancers	Blood, PBMC, lymphoblastoid cells, HUVEC, leukocytes	Exonic SNPs change predicted *ANRIL* free energy calculation, may impact secondary structure. Most intronic SNPs decrease *ANRIL* expression; possible predicted enhancers	(4, 14, 43, 48, 50, 69, 70)
	Exon 2	rs10965215				
	Intron 3	rs2151280				
	Exon 6	rs10738605				
	Intron 6	rs944799				
E	Intron 13	rs10116277 rs6475606 rs10738607 rs10757274	CAD, stroke, intracranial aneurysm, MI, endo- metriosis, hypertension, cancers	Blood, PBMC, PBTL, VSMC, atherosclerotic plaque, primary vascular tissue, lympho-blastoid, HUVEC	Isoform-specific *ANRIL* up/downregulation. Experimentally tested enhancer regions. rs10757278 may impact *ANRIL* splicing, promoting circ*ANRIL* production	(8, 14, 16, 43, 44, 48, 49, 51, 71–73)
	Intron 14	rs10757278				
	Intron 18	rs2383206 rs2383207				
	Intron 19	rs1333045				
	Distal to exon 21	rs10811656 rs1333049				
F	Distal to exon 21	rs2383208 rs10811661	Type 2 diabetes	Blood, islets	Decrease/increase *ANRIL* expression, Predicted enhancer region	(48, 68)
						

*Groups A–F are defined loosely based on linkage disequilibrium (defined as LD>0.8 in Caucasian population in LDHap) and by predicted or tested impact on ANRIL expression or structure. Intron and exon numbers are based on 21 exons*.

### Disease-associated SNPs may influence *ANRIL* abundance

There is no consistent global pattern with respect to SNP impact on *ANRIL* abundance. For most SNP groups, risk-SNPs are reported that both increase and decrease *ANRIL* levels in different studies. Variability may be related to differences in technique used to detect *ANRIL* that favor one isoform over others, cell type studied, acute and chronic biology and genetic origin of the cellular material studied, and of course the individual biology of each polymorphism. Most *ANRIL* SNPs fall in large linkage blocks, which are variable among different human genetic groups; in many cases the SNP tested may not be the causative SNP in the linkage block, and published linkage blocks may not apply to the material tested if not carefully matched by origin. It is entirely possible that all conflicting results are correct; for example, a CAD risk-SNP could increase pro-proliferative *ANRIL* isoforms in endothelial, macrophage or vascular smooth muscle cells to drive atherosclerosis, whereas a diabetes risk-SNP at the same position could decrease proliferative *ANRIL* isoforms in beta cells to limit beta cell mass. The complexity of the human system necessitates testing the relevant *ANRIL* isoforms in the relevant cell type, preferably in primary cells, in tissue- and disease-specific manner.

A comprehensive review of all SNP effects is beyond the scope of this review. Some *ANRIL* located disease-associated SNPs impact both *ANRIL* expression and *CDKN2A/CDKN2B* expression (14, 48); others impact *ANRIL* but not *CDKN2A* or *CDKN2B* (14, 48, 60), and still others impact *CDKN2A/CDKN2B* but not *ANRIL* (48, 50, 64). Some SNPs are located within predicted or proven enhancer regions (10, 43, 48, 49, 64, 75) or miRNA binding sites (65, 66), providing possible mechanisms of cell type specific gene regulation.

### Disease-associated SNPs may influence *ANRIL* structure or function

Beyond regulation of *ANRIL* transcription, polymorphisms could impact *ANRIL* function by influencing relative abundance of different isoforms through RNA splicing or stability, or through altering the secondary structure or interactions of any given isoform. Several studies have identified *ANRIL* isoform-specific effects (50, 53, 67, 71, 72); for example, four SNPs forming an atherosclerosis risk haplotype were associated with increased expression of some, but not all, *ANRIL* isoforms (44). SNPs may influence the relative abundance of linear compared to circular isoforms (8). Several SNPs are reported to impact *ANRIL* free energy of folding, resulting in a predicted change in secondary structure, with implications for function and stability (48, 69, 74).

## *ANRIL* in cancer

*ANRIL* was initially identified in a kindred of familial melanoma-neural system tumor with a germ-line deletion of the entire *CDKN2A/B* locus (2). Although the *CDKN2A/B* locus is deleted or silenced in approximately 40% of human cancers, related to the tumor suppressive actions of *CDKN2A* and *CDKN2B* (76), *ANRIL* itself has pro-oncogenic properties. *ANRIL* is implicated in many malignancies, including cancers of the bladder (77), ovary (78, 79), lung (38, 58, 80–82), liver (40, 54, 83), stomach (22), breast (57, 84, 85), esophagus (86), nasopharyngeal cavity (39, 87, 88), thyroid (89), bone (90), cervix (91), colon (92), prostate (21, 56), glioma (55), and others (76). High tissue abundance of *ANRIL* in cancers is associated with aggressive clinicopathologic features such as high histological grade tumor size, advanced tumor-node-metastasis stage, and poor overall survival (22, 38, 40, 58, 78, 79, 83, 87, 89, 91–93). Certain SNPs within the *ANRIL* gene are associated with *ANRIL* and *CDKN2A/B* locus gene expression and clinical parameters (4, 48, 70, 94–96). *ANRIL* may be useful as a prognostic biomarker and a therapeutic target for clinical cancer management.

### Molecular mechanisms of *ANRIL* in cancer

Accumulating evidence suggests that *ANRIL* participates in tumorigenesis by influencing cell proliferation, apoptosis and metastasis. Depletion or overexpression of *ANRIL* changes expression levels of many genes involved in proliferation, cellular adhesion and apoptosis (14, 32, 46). *ANRIL* overexpression promotes proliferation, migration, invasion, and epithelial-mesenchymal transformation but inhibits cell apoptosis; *ANRIL* loss-of-function represses tumor size and growth rate, cell proliferation, migration, invasion, metastasis, and enhances apoptosis and senescence (22, 38, 55, 56, 58, 77, 80, 81, 84, 89–92). Suppression of *ANRIL* is required for Ras-induced senescence (13, 21, 42). High *ANRIL* levels are associated with resistance to chemotherapy, and *ANRIL* knockdown may promote chemosensitivity (37, 79, 88, 97–99). On the other hand, *ANRIL* mediated anti-oncogenic effects of phospholipase D in lung cancers (82).

*ANRIL* may promote carcinogenesis through a number of mechanisms. Canonical *ANRIL* transcriptional mechanisms may play a role, such as by *in cis* suppression of the *CDKN2A/CDKN2B* tumor suppressor genes (80, 81, 100), or through PRC-mediated *in trans* gene regulation (40, 58, 80). *ANRIL* miRNA regulation has been implicated in cancers as well, including mechanisms involving let-7a and miR-125a in nasopharyngeal and oral carcinoma (56, 88, 101), miR-99a/miR-449a in gastric cancer (22), miR-122-5p in hepatocellular carcinoma (54), miR-186 in cervical cancer (91), and miR-199a in breast cancer (57), miR-34a in glioma (55), and miR-323 in pediatric medulloblastoma (102). Transcription factors affected by *ANRIL* in cancers include KLF2 (40, 58), SMAD (56, 86, 89) and β-catenin. *ANRIL* interacts with signal transduction pathways in cancers such as PI3K/AKT, p38 MAPK, TGF-β, ATM-E2F1, and MTOR (33, 55, 56, 86, 89, 99, 103). *ANRIL* can also drive cancer progression by increasing glucose uptake for glycolysis (87), through lymphangiogenesis via LYVE-1, VEFG-C, and VEGFR-3 (92), and through invasion and metastasis via MET and MMP3 (78). An intriguing but mostly unexplored phenomenon is breakpoint fusion transcripts including exons from *ANRIL* fused with exons from *MTAP*, a neighboring protein-coding gene, which were identified in 20% of screened melanoma cell lines (104).

## *ANRIL* in metabolic disease

In addition to cancer, genome-wide association studies have repeatedly and confidently identified links between the genomic region containing *ANRIL* and risk of developing cardiometabolic disease, including type 2 diabetes and manifestations of atherosclerosis such as CAD and stroke (10, 62). This locus influences risk not only of classic type 2 (obesity-related) diabetes, but also with related syndromes such as gestational diabetes, transplant-associated diabetes, and cystic fibrosis related diabetes, but not risk of type 1 (autoimmune) diabetes (10). Although diabetes is a clinical risk factor for atherosclerosis, the genetic influence for these conditions at the *ANRIL* locus is mostly non-overlapping, with atherosclerosis SNPs located throughout the *ANRIL* gene, and T2D SNPs located distal to the last *ANRIL* exon (7). One exception is a SNP located in *ANRIL* exon 2, rs564398, which is associated with both T2D and CAD (105). Since *CDKN2A/B* locus genes are known for their roles in cell cycle regulation and cancer, and not metabolism, many questions remain as to how this locus impacts metabolic disease.

### *ANRIL* and atherosclerotic disease

Since *ANRIL* locus SNPs influence risk of atherosclerosis, many studies have now tested whether *ANRIL* gene expression is related to atherosclerosis-associated diseases. In subjects with angiographically confirmed CAD in the Leipzig heart study, specific *ANRIL* isoforms were positively correlated with CAD risk SNP haplotype in PBMCs, whole blood, and atherosclerotic plaque tissue (44). In the Framingham heart study, *ANRIL* SNPs were associated with multiple CAD-related outcomes, and showed isoform-specific *ANRIL* correlation in leukocytes, with short isoforms predicted to contribute to CAD pathogenesis (50). CAD risk-SNPs may regulate the relative abundance of linear and circular *ANRIL* isoforms (8). Intriguingly, abundance of *ANRIL* in circulating plasma was positively correlated with in-stent restenosis (53), but in PBMCs harvested at the time of angioplasty/reperfusion, *ANRIL* levels were lower in subjects with myocardial infarction, but higher in subjects with older age, diabetes, hypertension. In this cohort, *ANRIL* levels in PBMCs improved model prediction of subsequent left ventricular dysfunction (106). *ANRIL* promoter methylation may mediate an epigenetic influence on future cardiac risk; higher CpG methylation at birth was associated with higher pulse wave velocity, a marker for increased arterial stiffness indicating greater cardiovascular risk, at 9 years of age (31).

Mechanisms by which *ANRIL* impacts atherosclerotic disease remain debated. In aortic smooth muscle cells, knockdown of *ANRIL* using siRNA targeting exon 1 or exon 19 revealed altered gene expression networks impacting cell proliferation, apoptosis, extracellular matrix, and inflammation (14). Atherogenic gene expression networks were regulated by *ANRIL* via the Alu mechanism, in which Alu motifs target *ANRIL* to particular gene locations, recruiting PRC complexes and altering gene methylation status (46). *ANRIL* may impact risk of ischemic stroke by regulating the Caspase recruitment domain 8 (CARD8) gene in endothelial cells (52). A known CAD-associated miRNA, miR-92a, may mediate some *ANRIL* effects; *ANRIL* targets GATA2, MAP1B, and ARG1 were found to require miR-92a, placing this miRNA downstream of *ANRIL* for some atherogenic effects (69). Finally, *ANRIL* is related to inflammation: *ANRIL* is increased by pro-inflammatory factors NF-κ B and TNF-α in endothelial cells, and *ANRIL* was found to bind directly to the YY1 transcription factor to mediate TNF-a induction of cytokines IL-6 and IL-8 (26).

### *ANRIL* and obesity, bone mass, and estrogen signaling

Although GWAS studies do not suggest a link between *CDKN2A/B* locus SNPs and obesity risk in adult populations, intriguingly, *ANRIL* may be a genomic site of environmental epigenetic influence on obesity. The *ANRIL* promoter contains CpG methylation sites that are differentially regulated across samples. In human tissues taken at birth, lower CpG methylation in infancy predicted higher fat mass at 6 years of age, as well as increased bone size, mineralization and density (29, 30). *ANRIL* promoter methylation was also negatively correlated with BMI in contemporaneous samples of peripheral blood from adolescents and in adipose tissue from adults (29). Methylation of these CpG sites increased tissue abundance of *ANRIL* RNA, in a mechanism that might include increased activity of an estrogen response element. Functional studies in a liposarcoma cell line showed that transcription factor binding to an adjacent ERE was enhanced by methylation, and estradiol increased *ANRIL* expression (29).

### *ANRIL* and type 2 diabetes

Multiple SNPs in different linkage blocks at the *CDKN2A/B* locus are associated with T2D risk; evidence in human populations suggests these SNPs impact pancreatic islet mass or function (10). Despite the fact that the T2D risk SNPs are located in or near the *ANRIL* gene, the field has largely assumed the effect was mediated by the protein coding genes at the locus, due to extensive published work implicating p16INK4A in the regulation of beta cell mass (10). However, although studies have found no association between *CDKN2A/B* T2D SNPs and transcript level of *p14ARF, p15INK4B*, or *p16INK4A* in human islets (10, 107), an age-dependent positive association was identified between distal T2D risk-SNPs (group F in Table 1) and *ANRIL* expression (68). On the other hand, a T2D risk-SNP in *ANRIL* exon 2 (group C in Table 1) was associated with reduced *ANRIL* expression, again with no change in *p14ARF, p15INK4B*, or *p16INK4A* expression (14, 48); however, these studies were carried out in blood rather than islets. In human islets, this exon 2 SNP was shown to remove a CpG methylation site; risk allele was associated with reduced islet insulin content but no change in locus gene expression (108). Risk allele at this SNP was associated with impaired beta cell proliferation response to high glucose (68). In a study relevant to diabetic retinopathy, high glucose exposure increased *ANRIL* expression in human retinal epithelial cells (25). In *ANRIL* was found to increase expression of VEGF, a critical element of the neovascularization that is central to damage from retinopathy, via a mechanism involving PRC2 and miR200b (25, 109).

## Summary

Studies suggest the *ANRIL* lncRNA influences risk of a number of diseases, including many types of cancer as well as metabolic disease. Current understanding of *ANRIL* biology indicates the primary function of this lncRNA is to regulate gene expression, both locally at *CDKN2A/B* as well as across the genome, via mechanisms including chromatin modulation, transcription factor binding, and miRNA regulation. Knowledge concerning ANRIL function in cancers is more solid and advanced than for metabolic tissues. Mechanisms by which SNPs influence ANRIL abundance remain uncertain and require more study; how DNA methylation regulates ANRIL in cancers also will benefit from more study. Much remains to be learned about the structural complexity of *ANRIL*; how the various identified linear and circular isoforms impact tissue biology to modulate disease risk is mostly unknown. There is an urgent need for deeper understanding of how *ANRIL* isoforms modulate cellular function in human organs and tissues, and to explore the differing roles of ANRIL in cancer and metabolic disease. Given the advent of RNA therapeutics, and the broad disease relevance of *ANRIL*, it is possible that these studies may lead to future disease prevention and treatment.

## Author contributions

YK, C-HH, and LA wrote and revised the manuscript.

### Conflict of interest statement

The authors declare that the research was conducted in the absence of any commercial or financial relationships that could be construed as a potential conflict of interest.

## Abbreviations

CAD: coronary artery disease
CAC: coronary artery calcium
CDK: cyclin dependent kinase
circ*ANRIL*: circular *ANRIL*
circRNA: circular RNA
GWAS: genome-wide association study
lncRNA: long noncoding RNA
miRNA: microRNA
MI: myocardial infarction
MTAP: S-methyl-5′-thioadenosine phosphorylase
PBMC: peripheral blood mononuclear cell
PBTL: peripheral blood T-lymphocytes
PRC: polycomb repressive complex
SNP: single nucleotide polymorphism
VSMC: vascular smooth muscle cells.
